# Modulation of Lung Adenocarcinoma by Phosphorylated FOXN3‐Mediated Transcriptional Inactivation of p53

**DOI:** 10.1002/advs.76449

**Published:** 2026-07-07

**Authors:** Jinjin Yu, Jingyu Yu, Suhui Wang, Xinghong Hu, Lele Jin, Nan Wu, Yuhan Ni, Xueying Zhang, Chengling Zhao, Jihong Zhou, Yong Zhang, Baofa Sun, Hongxing Zhang, Chunfu Zheng, Song Guo Zheng, Wei Li, Xinxing Zhu

**Affiliations:** ^1^ Anhui Province Key Laboratory of Respiratory Tumor and Infectious Disease Department of Respiratory and Critical Care Medicine First Affiliated Hospital Bengbu Medical University Bengbu China; ^2^ Molecular Diagnosis Center Bengbu Medical University Bengbu China; ^3^ Research Center of Clinical Laboratory Science School of Laboratory Medicine Bengbu Medical University Bengbu China; ^4^ State Key Laboratory of Medicinal Chemical Biology Frontiers Science Center For Cell Responses College of Life Sciences Nankai University Tianjin China; ^5^ School of Psychology Xinxiang Medical University Xinxiang China; ^6^ Department of Microbiology Immunology & Infection Diseases University of Calgary Calgary Alberta Canada; ^7^ Department of Immunology the School of Cell and Gene Therapy Songjiang Research Institute and Songjiang Hospital Affiliated to Shanghai Jiao Tong Uniersity School of Medicine Shanghai China; ^8^ Innovative Medical Research Institute Bengbu Medical University Bengbu China; ^9^ Center For Clinical Medicine of Respiratory Disease (tumor) in Anhui Bengbu China

**Keywords:** FOXN3, invasion, lung adenocarcinoma, p53 signaling, phosphorylation

## Abstract

As a pivotal tumor suppressor, p53 plays a critical role in the progression of lung adenocarcinoma (LUAD). However, the mechanisms through which its interacting partners modulate p53 transcriptional activity remain poorly understood. In this study, we identified the transcription factor FOXN3 as a key partner that recruits p53 for transcriptional responses. FOXN3 directly interacts with p53, and the two factors exhibit extensive genome‐wide colocalization in both lung cancer cells and clinical tumor tissues, thereby co‐regulating the transcription of numerous genes. Notably, nearly all p53 point mutants with disrupted DNA‐binding capacity show markedly reduced association with FOXN3, underscoring the essential role of FOXN3 in facilitating p53 binding to DNA. Conditional knockout of FOXN3 promotes lung cancer cell survival, invasion, and tumorigenesis. Importantly, the regulation of p53 transcriptional activity by FOXN3 requires phosphorylation at the S83 and S85 sites. This phosphorylation induces the dissociation of FOXN3 from chromatin, thereby inhibiting p53 transcriptional recruitment and activation. Ablation of FOXN3 S83 and S85 phosphorylation impedes the progression of LUAD. Furthermore, increased phosphorylation of FOXN3 at S83 and S85 is observed in clinical lung tumor tissues and correlates with poor prognosis in patients with LUAD, highlighting its potential as a therapeutic target.

## Graphical abstract

In non‐tumorous lung tissues, FOXN3 promotes the transcriptional activation of p53 by facilitating its recruitment to target promoters, thereby suppressing lung tumorigenesis through activation of the p53 signaling pathway. Conversely, in lung adenocarcinoma tissues, hyperphosphorylated FOXN3 dissociates from the promoters of p53‐responsive genes and displays diminished binding to p53. This disruption blocks p53 transcriptional recruitment and consequently inactivates p53 signaling.

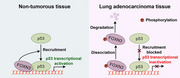

## 1 Introduction

Lung cancer is the leading cause of cancer‐related mortality and ranks as the second most frequently diagnosed cancer, following female breast cancer. Non‐small cell lung cancer (NSCLC) is the most common type, accounting for 80–85% of all lung tumors, with lung adenocarcinoma (LUAD) being the most prevalent subtype, accounting for approximately half of all NSCLC cases [1, 2]. Unfortunately, most lung cancer patients are diagnosed at an advanced, incurable stage, contributing significantly to the overall mortality from lung cancer. Therefore, a deeper understanding of the underlying mechanisms is crucial and urgent for identifying potential therapeutic targets for the treatment of LUAD. The tumor protein p53 (p53) serves as a vital tumor suppressor and participates in diverse biological processes, including cell cycle arrest, DNA repair, autophagy, apoptosis, immunity, senescence, ferroptosis, and metabolism [3, 4, 5, 6, 7, 8, 9, 10, 11]. As a transcription factor, its main role is to regulate approximately 500 target genes, including prominent examples such as CDKN1A (cyclin‐dependent kinase inhibitor 1, or p21), BBC3 (Bcl2‐binding component 3) and Bax (BCL2 associated X, an apoptosis regulator) [12, 13, 14, 15]. Through these functions, p53 is essential for inhibiting tumor development. Therefore, the regulation of p53 transcriptional activity is critical for lung cancer cell survival and tumorigenesis. In response to various stresses, p53 is rapidly activated and regulates numerous target genes at the transcriptional level, influencing a wide range of cellular activities. The transcriptional activity of p53 is precisely modulated by multiple cellular factors that can act either independently or synergistically to produce a specific transcriptional response [16, 17, 18, 19]. For example, p53 directly interacts with the coactivator p300/CBP to acetylate histones near p53 target promoters [20, 21], thereby enhancing its transcriptional response. Conversely, p53 can also recruit histone deacetylases (HDACs) to promoters by interacting with mSin3a, which directly binds to HDACs, leading to the transcriptional repression of target genes [22].

The forkhead box (FOX) family of transcription factors includes more than 50 members, which are phylogenetically categorized into 19 subclasses (A to S). All FOX proteins possess a homologous DNA‐binding domain known as the forkhead domain or winged helix [23, 24, 25]. These proteins play roles in a wide array of cellular processes, such as cell differentiation, proliferation, DNA repair, metabolism, and tumorigenesis [23, 24]. Nevertheless, the biological functions of mammalian forkhead transcription factors in the N class, including FOXN3, have yet to be thoroughly investigated. FOXN3 was originally described as a crucial transcriptional repressor. While FOXN3 reportedly interacts with xSin3/xRPD3 in *X. laevis* [26] and with Sin3 in *Saccharomyces cerevisiae* [27], as well as with MEN1 [28] and SKIP [29] in human cells to exert its transcriptional repressive function, it also activates transcription in the testes of Drosophila [30]. These findings indicate that the mechanistic role of FOXN3 in transcriptional regulation requires further investigation.

Numerous studies have indicated that FOXN3 is dysregulated in tumors of various tissue origins [31, 32, 33, 34, 35, 36, 37]. However, its specific role in tumorigenesis—particularly its contribution to the development and progression of LUAD—remains poorly understood. In this study, we identified FOXN3 as a critical transcriptional activator that recruits p53 to its DNA response elements for transcriptional activation through direct interaction. Loss of FOXN3 reduces the binding affinity of p53 to chromatin, rendering p53 functionally inactive and promoting lung cancer cell survival and tumorigenesis. Intriguingly, nearly all p53 mutants with reduced DNA‐binding capacity exhibit greatly suppressed association with FOXN3, highlighting the indispensable role of FOXN3 in modulating the DNA‐binding ability of p53 for gene transcriptional regulation. Elucidating the molecular basis by which FOXN3 regulates p53 transcriptional activity may open new avenues for cancer therapy.

## 2 Results

### 2.1 FOXN3 and p53 Exhibit Strong Colocalization at Gene Promoters

**FIGURE 1:**
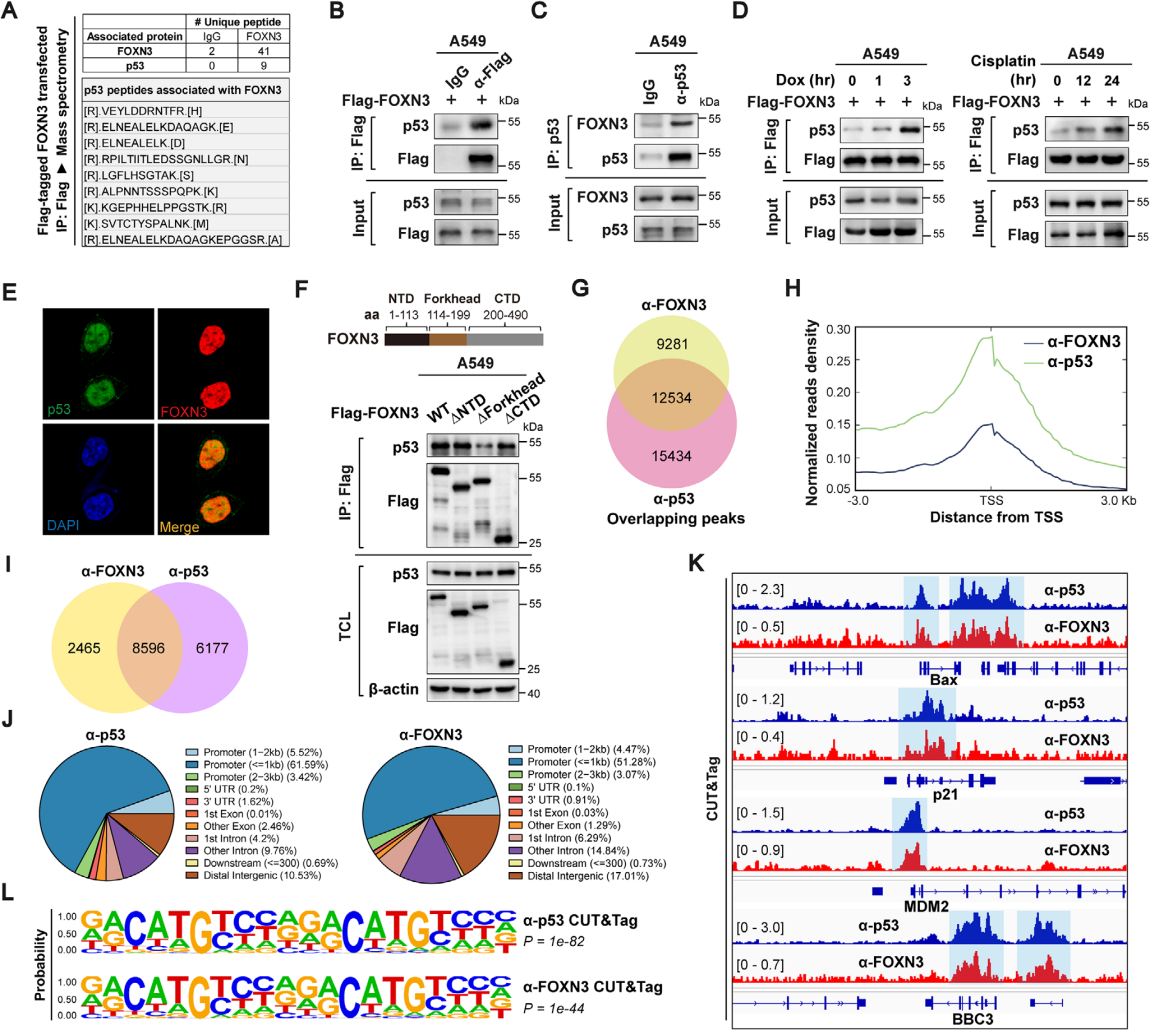
FOXN3 and p53 colocalize at the promoters of target genes. (A) The representative p53 peptides that were immunoprecipitated by Flag‐tagged FOXN3 were identified using mass spectrometry analysis. (B) An anti‐Flag Co‐IP assay was conducted in A549 cells to investigate the association of FOXN3 with endogenous p53. (C) An anti‐p53 Co‐IP assay was performed in A549 cells to investigate the endogenous association between endogenous FOXN3 and p53. (D) Anti‐Flag Co‐IP assays was performed in A549 cells to investigate the association of Flag‐tagged FOXN3 with endogenous p53 in the presence or absence of Dox (10 µm) or cisplatin (40 µg/mL) at the indicated time points. (E) An immunofluorescence assay was performed in A549 cells to assess the colocalization of FOXN3 and p53. (F) A deletion‐mapping assay was performed in A549 cells to define the region within FOXN3 associated with p53. (G) Venn diagrams showing the overlapping peaks from anti‐FOXN3 and anti‐p53 CUT&Tag analyses in A549 cells. (H) The density distributions of reads from the anti‐FOXN3 and anti‐p53 CUT&Tag analyses at the transcription start site (TSS) are displayed. (I) Venn diagrams illustrating the overlapping genes co‐targeted by FOXN3 and p53 in A549 cells, as determined via CUT&Tag data analysis. (J) Genomic distribution of the transcriptional targets of FOXN3 and p53 in A549 cells, as determined via CUT&Tag data analysis. (K) The binding profiles of FOXN3 and p53 to representative transcriptional targets of p53 are displayed by the Integrative Genomics Viewer (IGV) tool. (L) A representative p53 binding motif that is specifically bound by FOXN3 is presented.

To identify potential binding partners of the transcription factor FOXN3, we reanalyzed our previously published mass spectrometry dataset (ProteomeXchange Consortium, PXD032864) [38]. Further analysis of these data revealed a probable association between FOXN3 and p53 (Figure 1A). To verify this interaction, we conducted exogenous and endogenous coimmunoprecipitation (Co‐IP) assays in A549 cells, respectively. These assays confirmed that FOXN3 physically interacts with p53 (Figure 1B,C). Notably, this association was significantly enhanced following treatment with doxorubicin (Dox) or cisplatin (Figure 1D), both of which are known to activate p53 signaling. Immunofluorescence assays conducted in A549 cells further confirmed the subcellular colocalization of FOXN3 and p53 within the nuclear compartment (Figure 1E and Figure S1A). In light of our previous reports indicating that FOXN3 is a chromatin‐associated protein, with chromatin‐free FOXN3 being nearly undetectable [38], we hypothesize that their interaction occurs in a chromatin‐associated manner. To test this hypothesis, we initially conducted a deletion‐mapping assay to identify the specific region of FOXN3 required for their interaction. Our Co‐IP assays demonstrated that the loss of the forkhead DNA‐binding domain of FOXN3 significantly disrupted its interaction with p53 (Figure 1F and Figure S1B). This supports the notion that the interaction between FOXN3 and p53 relies on chromatin. We subsequently conducted Cleavage Under Target and Tagmentation (CUT&Tag) analysis to examine the genomic distribution of both FOXN3 and p53. We characterized 21 815 peaks from the anti‐FOXN3 CUT&Tag experiments and 27 968 peaks from the anti‐p53 CUT&Tag experiments, identifying 12 534 overlapping peaks (Figure 1G). To our surprise, further analysis revealed that 8596 genes were co‐targeted by FOXN3 and p53 at their gene promoters, resulting in similar genomic landscapes and peak distributions (Figure 1H–J). Notably, FOXN3 and p53 exhibit strikingly similar peak locations on the promoters of representative target genes of p53 (Figure 1K). The specificity analysis of the binding motifs demonstrated that both FOXN3 and p53 exhibit a specific interaction with a shared p53‐specific binding motif (Figure 1L). Additionally, the Kyoto Encyclopedia of Genes and Genomes (KEGG) analysis of the anti‐FOXN3 and anti‐p53 CUT&Tag data revealed co‐enrichment of multiple signaling pathways (Figure S1C,D), further supporting the potential physical interaction and functional relationship between FOXN3 and p53.

### 2.2 FOXN3 is Required for p53 Transcriptional Activity

**FIGURE 2:**
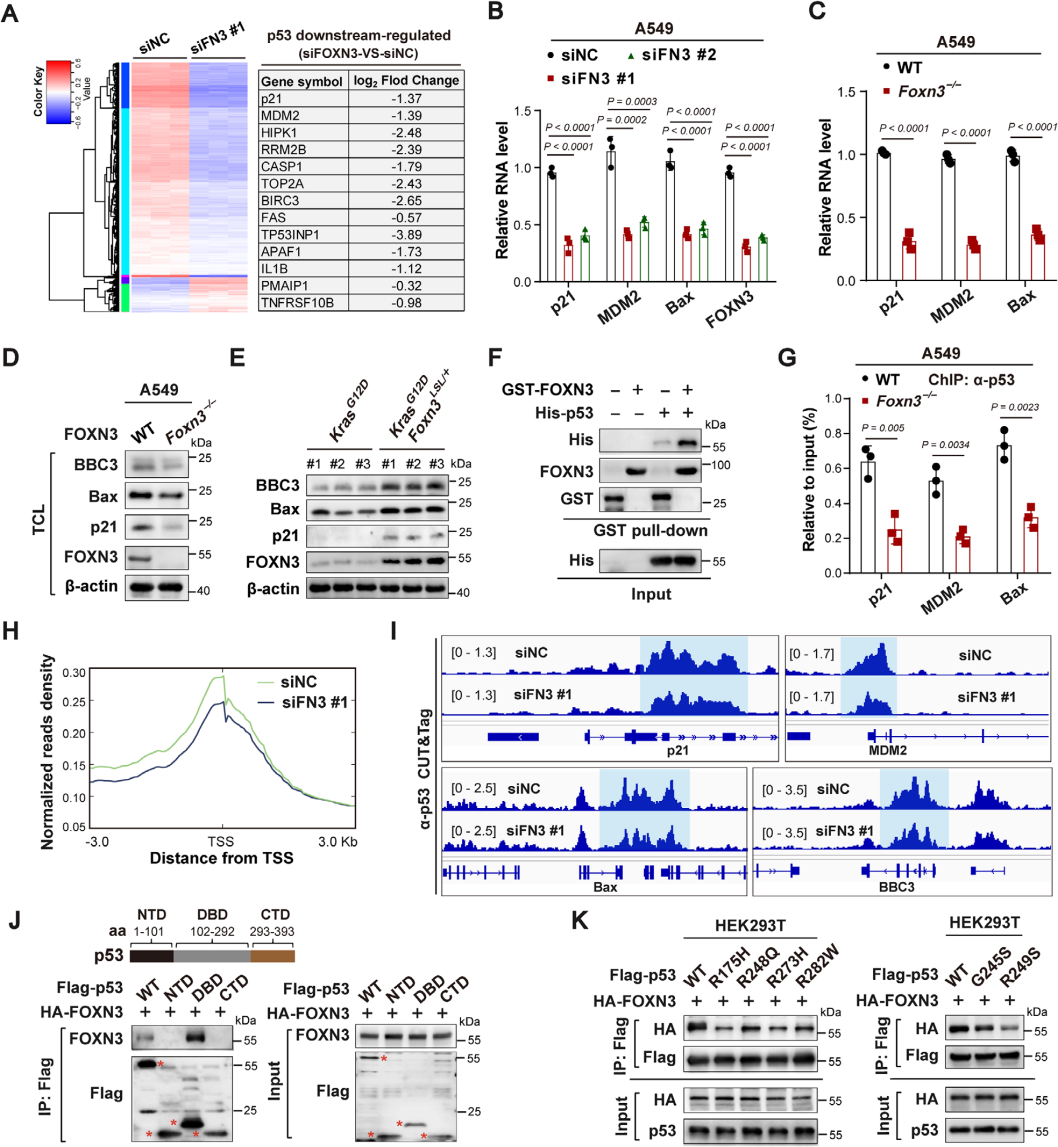
FOXN3 is essential for the transcriptional recruitment and activation of p53.(A) A heatmap was generated from mRNA sequencing analysis of A549 cells with and without FOXN3 knockdown to illustrate the changes in the expression of p53‐regulated genes. (B) A qPCR assay was performed in A549 cells with or without FOXN3 knockdown to examine the RNA levels of representative downstream target genes of p53.(C) A qPCR assay was performed in A549 cells to examine the effect of FOXN3 KO on RNA levels of representative p53 target genes. The cells were treated with Dox (10 µm, 3 h) prior to collection. (D) WB analysis was performed in WT and FOXN3‐KO A549 cells to examine the protein levels of representative downstream target genes of p53. The cells were treated with Dox (10 µm, 3 h) prior to collection. (E) Lung tumors isolated from *Kras^G12D/+^* & *Foxn3^LSL/+^* or *Kras^G12D/+^* mice were subjected to WB analysis to measure the levels of p53 transcriptional targets. (F) A GST pulldown assay was conducted in vitro to investigate the direct interaction between bacterially expressed FOXN3 and p53. (G) ChIP assays were conducted in WT and FOXN3‐KO A549 cells to evaluate the effect of FOXN3 depletion on the binding of p53 to the promoters of its target genes. The cells were treated with Dox (10 µm, 3 h) prior to collection. (H) Density distributions (read counts per million mapped reads) of p53 peaks at the transcription start site (TSS) in A549 cells, both with and without FOXN3 knockdown, are presented. (I) The binding profiles of p53 to its representative transcriptional targets in A549 cells, both with and without FOXN3 knockdown, are shown. (J) A deletion‐mapping assay was conducted in HEK293T cells to determine the specific region of p53 responsible for its interaction with FOXN3 (with p53 mutants indicated by an asterisk). (K) Immunoprecipitation assays were conducted in HEK293T cells to evaluate the interaction between Flag‐tagged p53 containing various point mutations and HA‐tagged FOXN3. Data B was assessed via one‐way ANOVA, and data C and G were assessed via a two‐tailed Student's *t*‐test. All the data are presented as the means ± SD.

The specific interaction and genome‐wide colocalization of FOXN3 and p53 prompted us to investigate the potential regulatory role of FOXN3 in modulating the p53 signaling pathway. To this end, we silenced FOXN3 in A549 cells and assessed the expression profile via mRNA sequencing. As anticipated, among the 2,148 downregulated and 347 upregulated genes, a subset of thirteen p53‐regulated genes exhibited a significant decrease following siRNA‐mediated FOXN3 depletion (Figure 2A). These findings were further validated through quantitative PCR (qPCR) and western blot (WB) analyses in both A549 and NIH3T3 cells, with and without FOXN3 knockdown or knockout (KO) (Figure 2B–D and Figure S2A–C). Additionally, FOXN3 overexpression enhanced the transcriptional response of p53 in a dose‐dependent manner, as determined by a luciferase assay (Figure S2D). To verify the regulatory effect of FOXN3 on p53 transcriptional activation in vivo, we utilized a loxP‐stop‐loxP (LSL) knock‐in (KI) mouse model harboring the Kras G12D mutation (*Kras^G12D/+^*). This model is widely used for studying LUAD associated with Kras G12D mutation [39]. We crossed this model with the FOXN3‐LSL KI strain (*Foxn3^LSL/+^*) that was previously generated [40] to assess the impact of FOXN3 overexpression on the levels of p53 target genes in tumors driven by the Kras G12D mutation. Following the intratracheal administration of an adeno‐associated virus (AAV) encoding Cre recombinase, we observed that aberrant overexpression of FOXN3 significantly elevated the protein levels of p53 response genes in lung tumors (Figure 2E).

To elucidate the molecular mechanism by which FOXN3 positively regulates p53 transcriptional activity, we first examined the impact of FOXN3 on p53 protein expression levels. WB analysis revealed that neither the knockout nor the knockdown of FOXN3 had any effect on p53 protein levels (Figure S2E,F). Next, we investigated the effect of FOXN3 on p53 nuclear translocation. Our fractionation and immunofluorescence assays indicated that FOXN3 knockdown does not impact p53 nuclear accumulation induced by Dox treatment (Figure S3A–C). We subsequently examined whether FOXN3 is involved in recruiting p53 to its target response elements for transcriptional activation. To investigate this, we first conducted a GST pulldown assay to examine the potential direct interaction between bacterially expressed FOXN3 and p53. WB analysis confirmed a direct binding interaction between the two proteins (Figure 2F). Next, we performed a chromatin immunoprecipitation (ChIP) assay to assess the impact of FOXN3 on the binding affinity of p53 to its transcriptional targets. Intriguingly, our results demonstrated that the knockout of FOXN3 significantly impaired the binding of p53 to its target gene promoters (Figure 2G). To further validate these findings, we performed a CUT&Tag assay to examine the genomic distribution of p53 in the presence or absence of FOXN3. This analysis consistently revealed a genome‐wide reduction in p53 occupancy at its response elements following the disruption of FOXN3 (Figure 2H). Additionally, visualization using the IGV tool confirmed a significant decrease in peak distribution at the promoters of representative p53 target genes following FOXN3 depletion (Figure 2I). Subsequently, we generated truncated mutations of p53 to identify the region responsible for its interaction with FOXN3. Notably, p53 interacted with FOXN3 through its DNA‐binding domain (Figure 2J). This observation, combined with previous findings indicating that the forkhead DNA‐binding domain of FOXN3 is responsible for its association with p53, suggests that FOXN3 is a crucial mediator required to recruit p53 for its binding to DNA response elements. To provide further evidence for this hypothesis, we generated several well‐known DNA‐binding‐deficient mutants of p53, specifically R175H, R248Q, R273H, R282W, G245S, and R249S. These mutations, located within the DNA‐binding domain, are well‐documented to impair p53's DNA‐binding capacity [41, 42, 43]. Surprisingly, we found that nearly all of the previously reported DNA‐binding‐deficient mutants exhibited a significantly reduced binding affinity for FOXN3 compared to WT p53 (Figure 2K). In summary, our findings suggest that FOXN3 is essential for the binding of p53 to its DNA response element through direct interaction, thereby facilitating the transcriptional activation of p53.

### 2.3 Disruption of FOXN3 Promotes Lung Cancer Cell Survival, Invasion, and Tumor Progression

**FIGURE 3:**
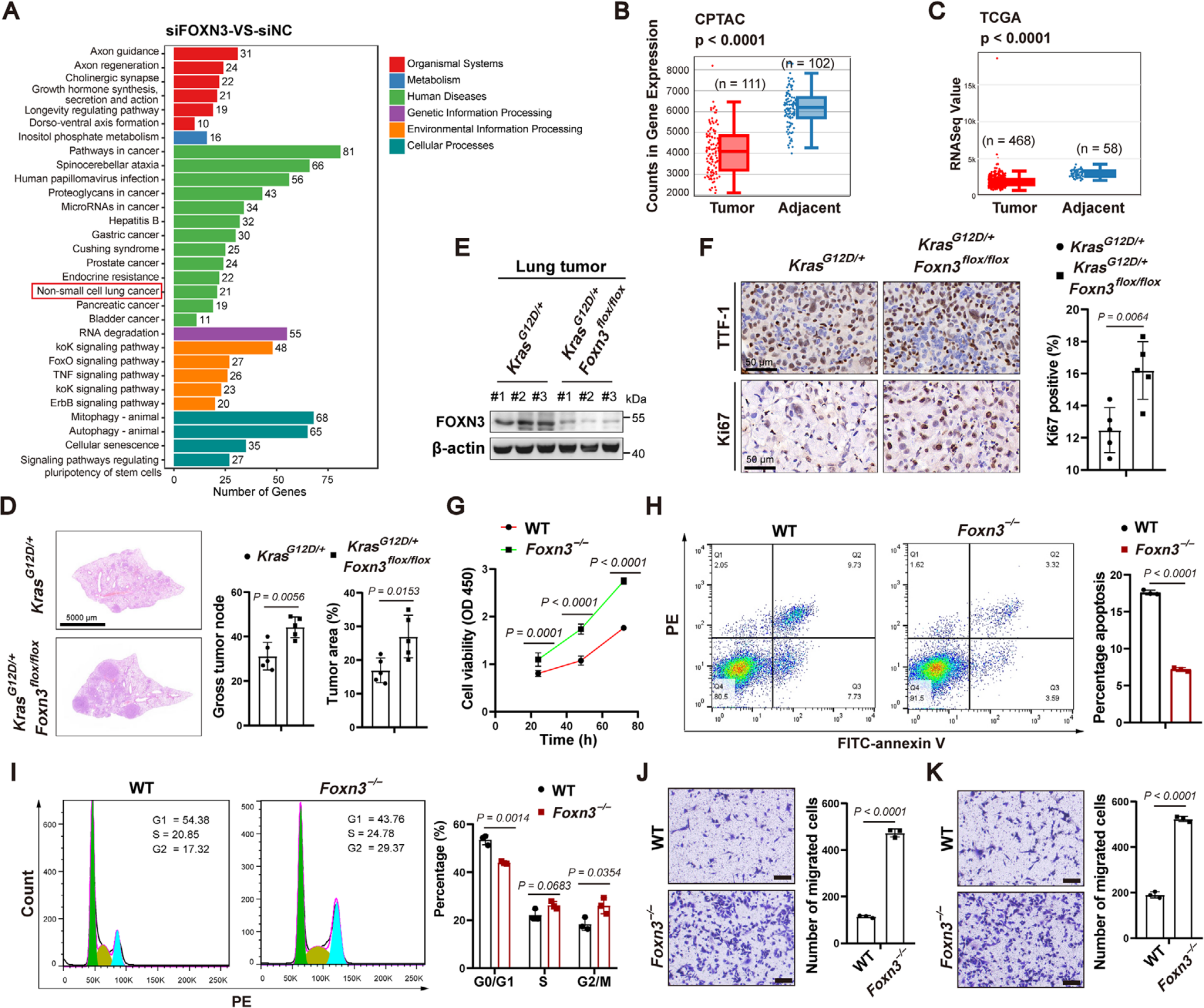
Disruption of FOXN3 promotes lung cancer cell survival, invasion and tumor progression. (A) Kyoto Encyclopedia of Genes and Genomes (KEGG) analysis revealed the pathways and biological processes enriched following FOXN3 knockdown in A549 cells. (B,C) Data mining of the CPTAC (B) and TCGA (C) databases revealed the RNA levels of FOXN3 in tumor and adjacent normal tissues of patients with LUAD.(D) H&E staining analysis of lung sections from *Kras^G12D/+^* & *Foxn3^flox/flox^* or *Kras^G12D/+^* mice that were administered AAV‐Cre for 18 weeks. The lung tumor burden was quantified via ImageJ software (*n* = 5). (E) WB analysis of lung tumors from *Kras^G12D/+^* & *Foxn3^flox/flox^* or *Kras^G12D/+^* mice that were administered AAV‐Cre for 18 weeks.(F) Anti‐TTF‐1 and ‐Ki67 immunohistochemical staining analysis of lung sections from *Kras^G12D/+^* & *Foxn3 ^flox/ flox^* or *Kras^G12D/+^* mice (*n* = 5) that were administered AAV‐Cre for 18 weeks. (G) The viability of A549 cancer cells was assessed using a Cell Counting Kit‐8 (CCK‐8) assay, both with and without FOXN3 knockout. (H) An apoptosis assay was conducted in A549 cells to investigate the effect of FOXN3 knockout on lung cancer cell apoptosis. The cells were treated with Dox (500 nm, 24 h) prior to collection. (I) Cell cycle analysis was conducted in A549 cells to investigate the effect of FOXN3 knockout on lung cancer cell cycle progression. The cells were treated with Dox (500 nm, 6 h) prior to collection. (J and K) Invasion (J) and migration (K) assays were performed in A549 cells with or without FOXN3 knockout to assess the effect of FOXN3 on cancer cell invasion. The cells were treated with Dox (500 nm, 6 h) prior to collection. Scale bar, 100 µm. The data D, F, and H‐K were assessed via two‐tailed Student's *t‐*tests, and the data G was assessed via two‐way ANOVA. All the data are presented as the means ± SD.

As a key tumor suppressor gene, p53 is functionally involved in the regulation of LUAD [44]. We therefore investigated whether FOXN3 plays a role in the progression of this cancer. To test this hypothesis, we conducted a further analysis of the mRNA sequencing data and found that FOXN3 is closely associated with non‐small cell lung cancer (Figure 3A). Notably, data from both the Clinical Proteomic Tumor Analysis Consortium (CPTAC) of the National Cancer Institute and the Cancer Genome Atlas revealed a significant reduction in FOXN3 RNA expression in LUAD tumor tissues compared with adjacent normal samples (Figure 3B,C).

To evaluate the potential inhibitory role of FOXN3 in LUAD, we crossed FOXN3 Flox (flanked by loxP) mice (*Foxn3^flox/flox^*) with *Kras^G12D/+^* mice to obtain double‐mutant strain. Subsequently, we administered AAV encoding Cre recombinase (AAV‐Cre) to assess the impact of FOXN3 knockout on primary lung tumor formation induced by the Kras G12D mutation. Interestingly, ablation of FOXN3 accelerated lung tumor progression (Figure 3D,E). Consistent with these findings, the cell proliferation marker Ki67 was significantly elevated in lung tumors following FOXN3 deletion compared with *Kras^G12D/+^* control mice (Figure 3F, lower panel), further confirming the crucial inhibitory role of FOXN3 in lung tumorigenesis in vivo. Additionally, the specificity of LUAD was characterized by anti‐TTF‐1 immunostaining (Figure 3F, upper panel). To confirm the suppressive role of FOXN3 in the development of LUAD, we employed NOD/SCID (severe combined immunodeficiency) mice to establish another widely used lung cancer model via the transplantation of A549 lung cancer cells. We simultaneously transplanted FOXN3‐depleted A549 cells and control‐treated A549 cells into the mice via tail vein injection. Six weeks post‐injection, we evaluated lung tumor burden using H&E staining analysis. Our results demonstrated that the transplantation of FOXN3‐depleted A549 cells significantly accelerated lung tumorigenesis compared to the control A549 cells (Figure S4A–C).

We subsequently examined the regulatory role of FOXN3 in lung cancer and NIH3T3 cell survival and invasiveness in vitro. Interestingly, both FOXN3 knockout and knockdown enhanced the viability of A549 and NIH3T3 cells, respectively (Figure 3G and Figure S4D). Consistent with this finding, apoptosis assays showed that the disruption of FOXN3 resulted in a reduced percentage of apoptotic cells (Figure 3H and Figure S4E). In line with it, the results of the cell cycle assays indicated that FOXN3 deficiency enhanced lung cancer and NIH3T3 cell cycle progression (Figure 3I and Figure S4F). We then assessed the impact of FOXN3 on cell invasion, and our results demonstrated that the loss of FOXN3 facilitated invasion and migration in both lung cancer and NIH3T3 cells (Figure 3J,K and Figure S4G,H). On the basis of these findings, we conclude that disruption of FOXN3 promotes cell survival, invasion, migration, and tumor progression.

### 2.4 Conditional Overexpression of FOXN3 Impedes Lung Cancer Cell Survival, Invasion and Tumor Progression

**FIGURE 4:**
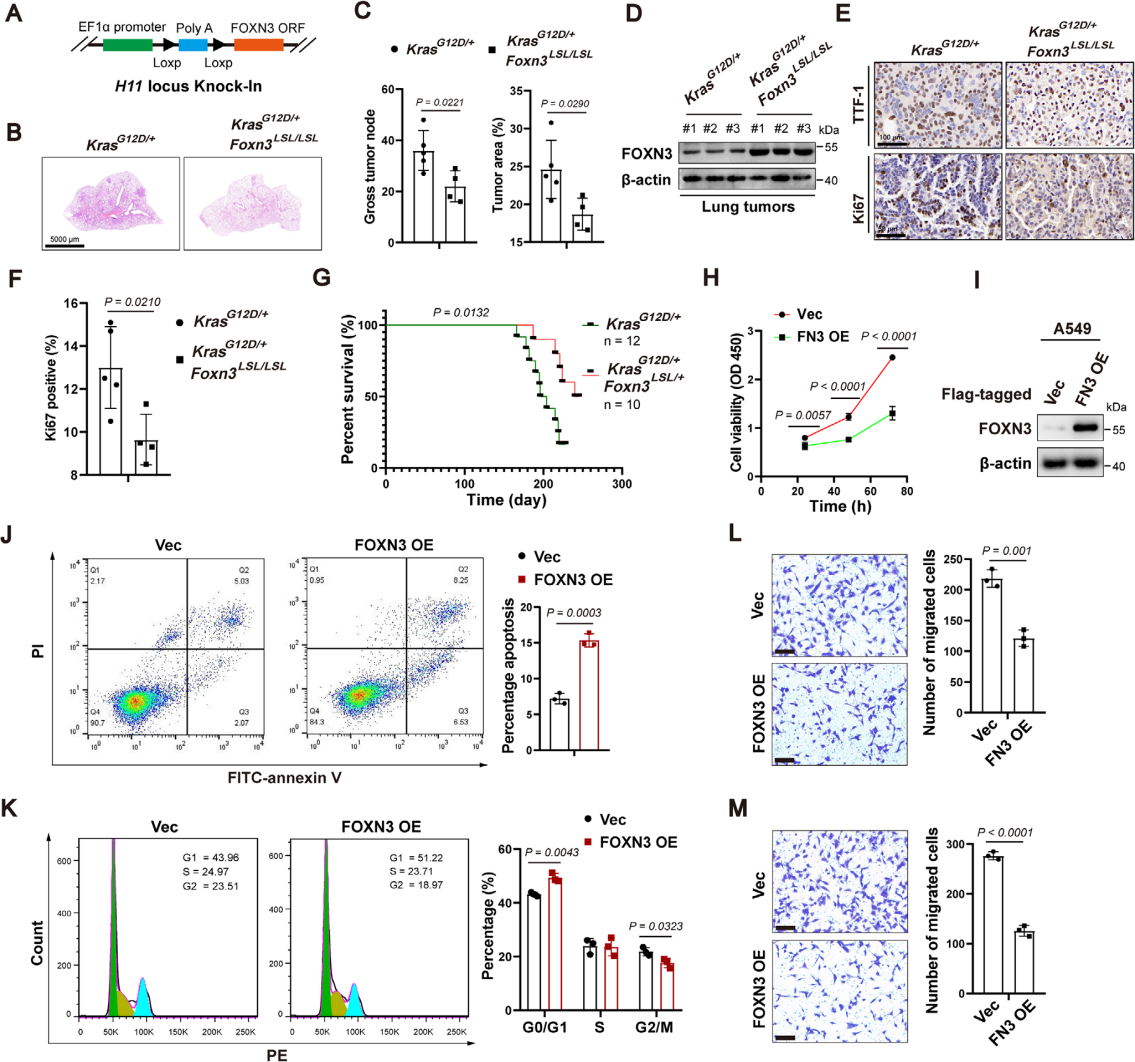
Aberrant FOXN3 overexpression impedes lung cancer cell survival, invasion and tumor formation. (A) A schematic diagram illustrating the generation of *Foxn3^LSL^* KI mice. (B and C) H&E staining analysis of lung sections from *Kras^G12D/+^* & *Foxn3^LSL/LSL^* or *Kras^G12D/+^* mice (*n* = 5 for *Kras^G12D/+^* group and *n* = 4 for *Kras^G12D/+^* & *Foxn3^LSL/LSL^* group) that were administered AAV‐Cre for 18 weeks. The lung tumor burden was quantified via ImageJ software. (D) WB analysis of lung tumors from *Kras^G12D/+^* & *Foxn3^LSL/LSL^* or *Kras^G12D/+^* mice was performed to assess the protein levels of FOXN3. The mice were administered AAV‐Cre for 18 weeks. (E and F) Anti‐Ki67 and ‐TTF‐1 immunohistochemical staining analysis of lung tumors from *Kras^G12D/+^* & *Foxn3^LSL/LSL^* or *Kras^G12D/+^* mice (*n* = 5 for *Kras^G12D/+^* group and *n* = 4 for *Kras^G12D/+^* & *Foxn3^LSL/LSL^* group). The Ki67‐positive cells were quantified via ImageJ software. The mice were administered AAV‐Cre for 18 weeks. (G) Kaplan–Meier survival curves of *Kras^G12D/+^* & *Foxn3^LSL/LSL^* or *Kras^G12D/+^* mice. (H) The viability of A549 cancer cells was assessed using a CCK‐8 assay, both with and without FOXN3 overexpression. (I) WB analysis of total lysates from A549 cells with and without FOXN3 overexpression via lentiviral infection. (J) An apoptosis assay was conducted in A549 cells to investigate the effect of FOXN3 overexpression on lung cancer cell apoptosis. The cells were treated with Dox (500 nm, 6 h) prior to collection. (K) Cell cycle analysis was conducted in A549 cells to investigate the effect of FOXN3 overexpression on lung cancer cell cycle progression. The cells were treated with Dox (500 nm, 6 h) prior to collection. (L,M) Invasion (L) and migration (M) assays were performed in A549 cells with or without FOXN3 overexpression to assess the effect of FOXN3 on cancer cell invasion. The cells were treated with Dox (500 nm, 6 h) prior to collection. Scale bar, 100 µm. The data C, F, and J–M were assessed via two‐tailed Student's *t*‐tests, and the data H was assessed via two‐way ANOVA. All the data are shown as the means ± SD.

To validate the suppressive role of FOXN3 in lung tumorigenesis, we additionally crossed FOXN3 loxp‐stop‐loxp knock‐in mice, which conditionally overexpress FOXN3, with *Kras^G12D/+^* mice to generate *Foxn3^LSL/+^& Kras^G12D/+^* double‐mutant strain. This allowed us to evaluate the effect of FOXN3 overexpression on lung tumor progression driven by the Kras G12D mutation. In line with the previous data, mice with conditional overexpression of FOXN3—achieved through the administration of an AAV encoding Cre recombinase—exhibited a reduced lung tumor burden compared to *Kras^G12D/+^* control mice, as determined by hematoxylin and eosin (H&E) staining analysis (Figure 4A–D). Furthermore, anti‐Ki67 immunohistochemical staining revealed decreased lung cancer cell proliferation upon FOXN3 overexpression (Figure 4E,F). We subsequently compared the survival rates of the *Foxn3^LSL/+^& Kras^G12D/+^* double‐mutant mice with those of the *Kras^G12D/+^* control group. As expected, the *Foxn3^LSL/+^ & Kras^G12D/+^* double‐mutant mice with forced FOXN3 overexpression displayed significantly improved survival rates compared to those of the *Kras^G12D/+^* mice (Figure 4G). We additionally investigated the functional involvement of FOXN3 in lung cancer cell survival and invasion in vitro. Consistent with the in vivo data, overexpression of FOXN3 suppressed cancer cell proliferation and promoted apoptosis in A549 cells (Figure 4H–J). Consistent with these apoptotic results, high levels of FOXN3 impeded lung cancer cell cycle progression (Figure 4K). We further evaluated the impact of FOXN3 overexpression on the invasive capabilities of lung cancer by performing in vitro invasion and migration assays. As anticipated, elevated FOXN3 expression led to a significant reduction in lung cancer cell invasion (Figure 4L,M). These collective findings strongly demonstrate that high levels of FOXN3 impede lung cancer cell survival, invasion, migration, and tumor formation.

### 2.5 Phosphorylation of FOXN3 at the S83 and S85 Sites Modulates the Transcriptional Activity of p53

**FIGURE 5:**
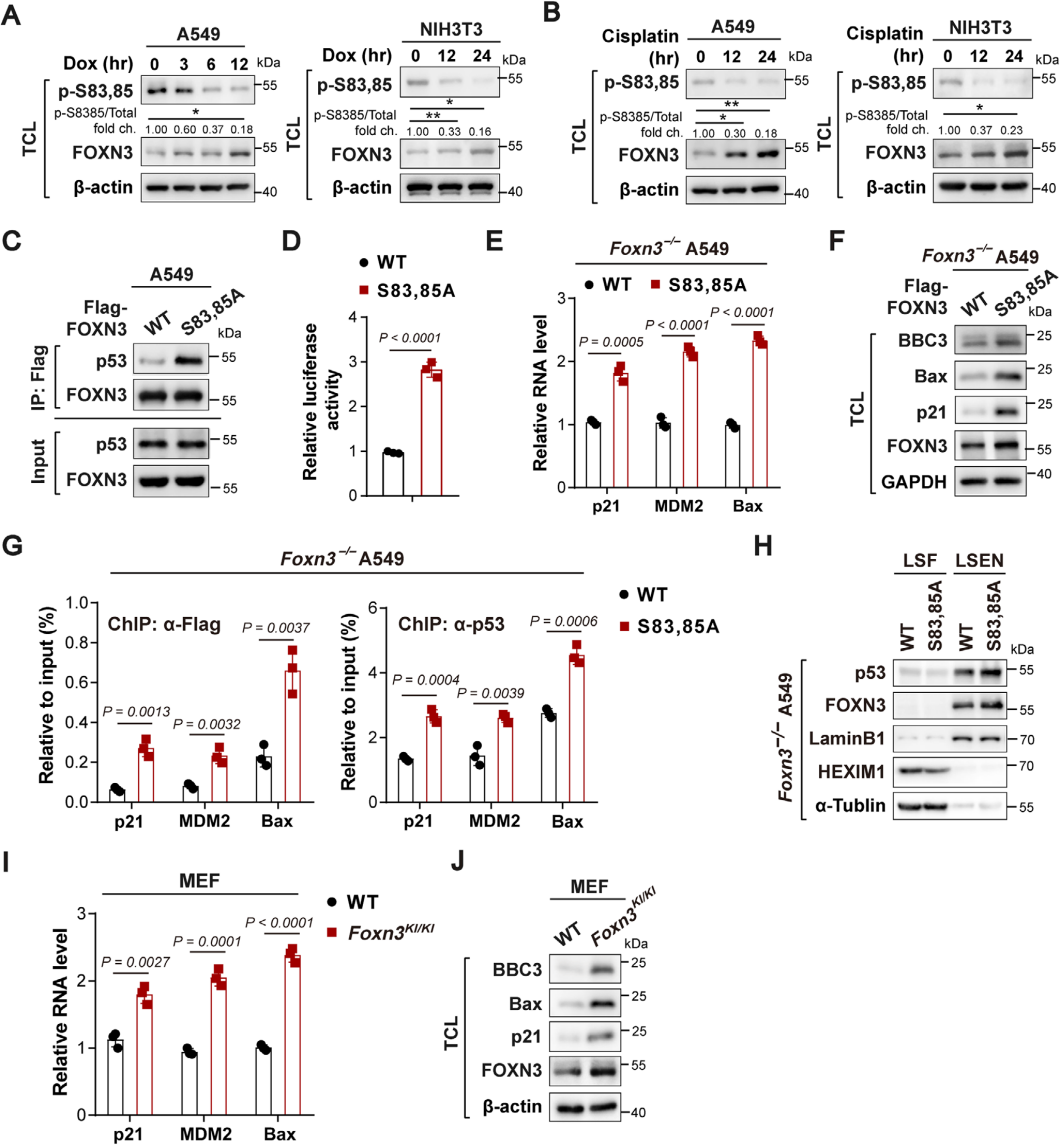
Blocking the phosphorylation of FOXN3 at S83 and S85 enhances p53 transcriptional activity in lung cancer cells. (A) WB analysis of total lysates from A549 and NIH3T3 cells treated with Dox at the indicated time points. A549: 10 µm Dox; NIH3T3: 2 µm Dox. (B) WB analysis of total lysates from A549 and NIH3T3 cells treated with cisplatin (40 µg/mL) at the indicated time points. (C) Co‐IP assays was performed in A549 cells expressing Flag‐tagged WT FOXN3, along with its S83,85A mutants, to assess their interactions with p53. The cells were treated with Dox (10 µm, 3 h) prior to collection. (D) A luciferase assay was conducted in *Foxn3^−/−^* A549 cells to assess the effects of Flag‐tagged WT FOXN3 or its S8385A mutant overexpression on p53 transcriptional activity. The cells were treated with Dox (10 µm, 3 h) prior to collection. (E) qPCR analysis was conducted on *Foxn3^−/−^* A549 cells infected with a lentivirus expressing flag‐tagged WT or S83,85A mutant of FOXN3 to assess the effect of blocking FOXN3 phosphorylation at S83 and S85 on the RNA levels of p53 target genes. The cells were treated with Dox (10 µm, 3 h) prior to collection. (F) WB analysis was conducted on *Foxn3^−/−^* A549 cells infected with a lentivirus expressing flag‐tagged WT or S83,85A mutant of FOXN3 to assess the effect of blocking FOXN3 phosphorylation at S83 and S85 on the protein levels of p53 target genes. The cells were treated with Dox (10 µm, 3 h) prior to collection. (G) ChIP assays were performed on *Foxn3^−/−^* A549 cells infected with a lentivirus expressing flag‐tagged WT FOXN3 or the S83,85A mutant to assess the effect of blocking FOXN3 phosphorylation at S83 and S85 on the binding affinity of p53 to the promoters of its target genes. The cells were treated with Dox (10 µm, 3 h) prior to collection. (H) A fractionation assay was performed in *Foxn3^−/−^* A549 cells infected with a lentivirus expressing flag‐tagged WT FOXN3 or the S83,85A mutant to assess the effect of the abrogation of S83 and S85 phosphorylation on p53 binding to chromatin. (I) qPCR analysis was conducted on WT and *Foxn3^KI/KI^* MEFs to assess the effects of blocking FOXN3 phosphorylation at S83 and S85 on the RNA levels of p53 transcriptional targets. The cells were treated with Dox (10 µm, 3 h) prior to collection. (J) WB analysis was conducted on WT and *Foxn3^KI/KI^* MEFs to assess the effects of blocking FOXN3 phosphorylation at S83 and S85 on the protein levels of p53 transcriptional targets. The cells were treated with Dox (10 µm, 3 h) prior to collection. The data D, E, G, and I were assessed via a two‐tailed Student's *t*‐test. All the data are shown as the means ± SD.

Our previous reports demonstrated that the phosphorylation of FOXN3 at the S83 and S85 residues triggers its dissociation from chromatin, leading to subsequent degradation [38]. On the basis of our findings that FOXN3 colocalizes with and recruits p53 to its response elements for transcriptional response, we hypothesized that the phosphorylation of FOXN3 at S83 and S85 likely modulates p53 transcriptional activity. To test this hypothesis, we initially monitored the levels of phosphorylation under conditions that activate p53 signaling. Intriguingly, stimulation with either doxorubicin (Dox) or cisplatin resulted in decreased phosphorylation at S83 and S85 in a time‐dependent manner (Figure 5A,B). This was accompanied by a gradual increase in total FOXN3 protein levels (Figure 5A,B), suggesting that phosphorylation‐dependent degradation of FOXN3 at S83 and S85 may play a significant role in the transcriptional regulation of p53 signaling. We subsequently conducted a Co‐IP assay to evaluate the binding affinity of WT FOXN3 and its S83,85A and S83,85D mutant with p53. WB analysis revealed that the abrogation of FOXN3 S83 and S85 phosphorylation resulted in a significantly stronger interaction with p53 compared to WT FOXN3 (Figure 5C). Additionally, we performed a luciferase assay to assess the effects of FOXN3 S83 and S85 phosphorylation on p53 transcriptional activity. Our results indicated that the overexpression of the FOXN3 S83,85A mutant significantly increased p53 transcriptional activity compared to the WT FOXN3 (Figure 5D). In accordance with these data, the exogenous reintroduction of the FOXN3 S83,85A mutant, compared to its WT form, enhanced the transcriptional activation of p53 target genes in *Foxn3^−/−^* A549 cells (Figure 5E,F). In contrast, the phospho‐mimetic mutant S83,85D displayed a disrupted association with p53 (Figure S5A), resulting in impaired transcriptional activation of p53 (Figure S5B). Consistent with these findings, our ChIP assays demonstrated that the reintroduction of the S83,85A mutant resulted in a stronger association of p53 with its target gene elements compared to WT FOXN3 (Figure 5G). Furthermore, we observed a greater association of the S83,85A mutant of FOXN3 with the promoters of p53 target genes relative to its WT counterpart (Figure 5G), which aligns with our previous report indicating that the phosphorylation of FOXN3 at S83 and S85 promotes its dissociation from chromatin [45]. Additionally, a stepwise fractionation assay provided further support for this observation, demonstrating that the S83 and S85 mutations enhance FOXN3 binding to chromatin, leading to increased enrichment of p53 on chromatin (Figure 5H). To further investigate the transcriptional regulatory role of FOXN3 phosphorylation, we isolated primary mouse embryonic fibroblasts (MEFs) from knock‐in (KI) mice in which the S83 and S85 residues of FOXN3 were replaced with alanine (*Foxn3^KI/KI^*). We also assessed the levels of p53 target genes in both *Foxn3^KI/KI^* and WT MEFs. Consistently, our findings revealed that disruption of S83 and S85 phosphorylation resulted in increased p53 transcriptional activity (Figure 5I,J). In summary, these observations suggest that the phosphorylation of FOXN3 at S83 and S85 restrains the transcriptional activation of p53 signaling.

### 2.6 Ablation of FOXN3 S83 and S85 Phosphorylation Impedes Lung Cancer Cell Survival, Invasion and Tumor Formation

**FIGURE 6:**
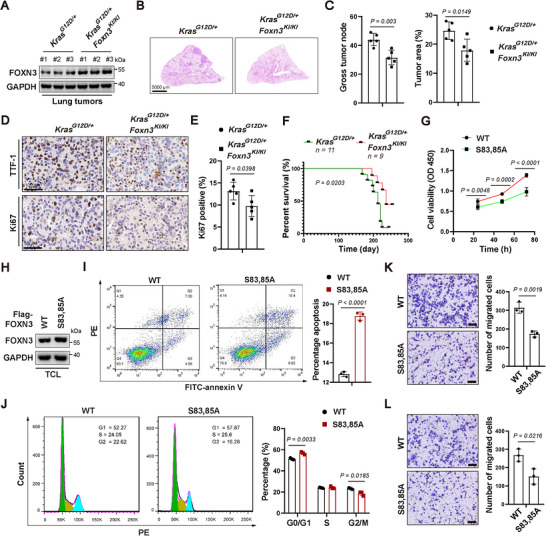
Ablation of FOXN3 S83 and S85 phosphorylation promotes lung cancer cell survival, invasion and tumor formation. (A) Western blot analysis was performed to assess the protein levels of FOXN3 in lung tumors isolated from *Kras^G12D/+^* & *Foxn3^KI/KI^* or *Kras^G12D/+^* mice that were administered AAV‐Cre for 18 weeks. (B,C) H&E staining analysis of lung sections from *Kras^G12D/+^* & *Foxn3^KI/KI^* or *Kras^G12D/+^* mice that were administered AAV‐Cre for 18 weeks. The lung tumor burden was quantified via ImageJ software. (D,E) Anti‐Ki67 and ‐TTF‐1 immunohistochemical staining analysis of lung tumors from *Kras^G12D/+^* & *Foxn3^KI/KI^* or *Kras^G12D/+^* mice. The Ki67‐positive cells were quantified via ImageJ software. The mice were administered AAV‐Cre for 18 weeks. (F) Kaplan–Meier survival curves of *Kras^G12D/+^* & *Foxn3^KI/KI^* or *Kras^G12D/+^* mice. (G) The viability of *Foxn3^−/−^* A549 cancer cells overexpressing Flag‐tagged WT FOXN3 or its S83,85A mutant were assessed using a CCK‐8 assay. (H) WB analysis was performed to assess the levels of WT FOXN3 or its S83,85A mutant in *Foxn3^−/−^* A549 cells. (I) An apoptosis assay was conducted in *Foxn3^−/−^* A549 cells overexpressing Flag‐tagged WT FOXN3 or its S83,85A mutant to investigate the effects of FOXN3 S83 and S85 phosphorylation on lung cancer cell apoptosis. The cells were treated with Dox (500 nm, 6 h) prior to collection. (J) Cell cycle analysis was conducted in *Foxn3^−/−^* A549 cells overexpressing Flag‐tagged WT FOXN3 or its S83,85A mutant to investigate the effects of FOXN3 S83 and S85 phosphorylation on lung cancer cell cycle progression. The cells were treated with Dox (500 nm, 6 h) prior to collection. (K,L) Invasion (K) and migration (L) assays were performed in *Foxn3^−/−^* A549 cells overexpressing WT FOXN3 or its S83,85A mutant to assess the effect of FOXN3 phosphorylation on cancer cell invasion. The cells were treated with Dox (500 nm, 6 h) prior to collection. Scale bar, 100 µm. The data C, E, and I–L were assessed via two‐tailed Student's *t*‐tests, and the data G was assessed via two‐way ANOVA. All the data are shown as the means ± SD.

Since phosphorylation of FOXN3 at S83 and S85 is implicated in the modulation of p53 signaling activity, we sought to determine whether this phosphorylation event also affects LUAD. To investigate this, we crossed the *Foxn3* knock‐in (KI) mice, in which both serine residues at the S83 and S85 sites were genetically substituted with alanine [46], with *Kras^G12D/+^* mice to generate double‐mutant strain (*Foxn3^KI/KI^* & *Kras^G12D/+^*). As shown in Figure 6A, inhibiting FOXN3 S83 and S85 phosphorylation resulted in an increase in FOXN3 protein levels, which aligns with our previous findings that phosphorylation at these sites triggers FOXN3 instability [38]. H&E staining analysis indicated that disruption of FOXN3 S83 and S85 phosphorylation significantly reduced the lung tumor burden (Figure 6B,C). Furthermore, the lung tumors from the *Foxn3^KI/KI^* & *Kras^G12D/+^* mice exhibited decreased lung cancer cell proliferation compared with those from the *Kras^G12D/+^* control mice, as assessed by anti‐Ki67 immunohistochemical staining (Figure 6D,E). We subsequently investigated the impact of FOXN3 phosphorylation on the survival rates of LUAD in mice. Notably, the *Foxn3^KI/KI^* & *Kras^G12D/+^* mice demonstrated improved survival rates compared with the *Kras^G12D/+^* mice (Figure 6F), suggesting that inhibiting FOXN3 S83 and S85 phosphorylation enhances survival by suppressing the progression of Kras G12D mutation‐driven lung tumor formation. Consistent with these observations, our NOD/SCID lung cancer model clearly indicated that the transplantation of A549 cells overexpressing the phosphorylation‐blocked S83,85A mutant exhibited reduced lung tumor formation compared to those overexpressing WT FOXN3 (Figure S6A–C), further validating the critical role of FOXN3 phosphorylation at S83 and S85 in lung tumorigenesis.

To further confirm the inhibitory effect of disrupting FOXN3 S83 and S85 phosphorylation on lung cancer invasiveness in vitro, we performed reintroduction of either WT FOXN3 or its S83,85A mutant form in *Foxn3^−/−^* A549 cells through lentivirus infection. Consistent with our in vivo findings, inhibiting the phosphorylation of S83 and S85 resulted in decreased lung cancer cell viability and increased apoptosis compared to WT FOXN3 (Figure 6G–I). Cell cycle analysis revealed marked suppression of lung cell cycle progression after disruption of S83 and S85 phosphorylation (Figure 6J). Furthermore, assessments of cell invasive capacity revealed that lung cancer cells expressing the FOXN3 S83,85A mutant exhibited reduced invasion and migration compared to those expressing WT FOXN3 (Figure 6K,L). These collective findings suggest that suppressing FOXN3 S83 and S85 phosphorylation inhibits lung cancer cell survival, invasion, and tumor progression.

### 2.7 The Phosphorylation‐Mediated FOXN3/p53 Transcriptional Response is Closely Associated With the Prognosis of Patients With Clinical LUAD

**FIGURE 7:**
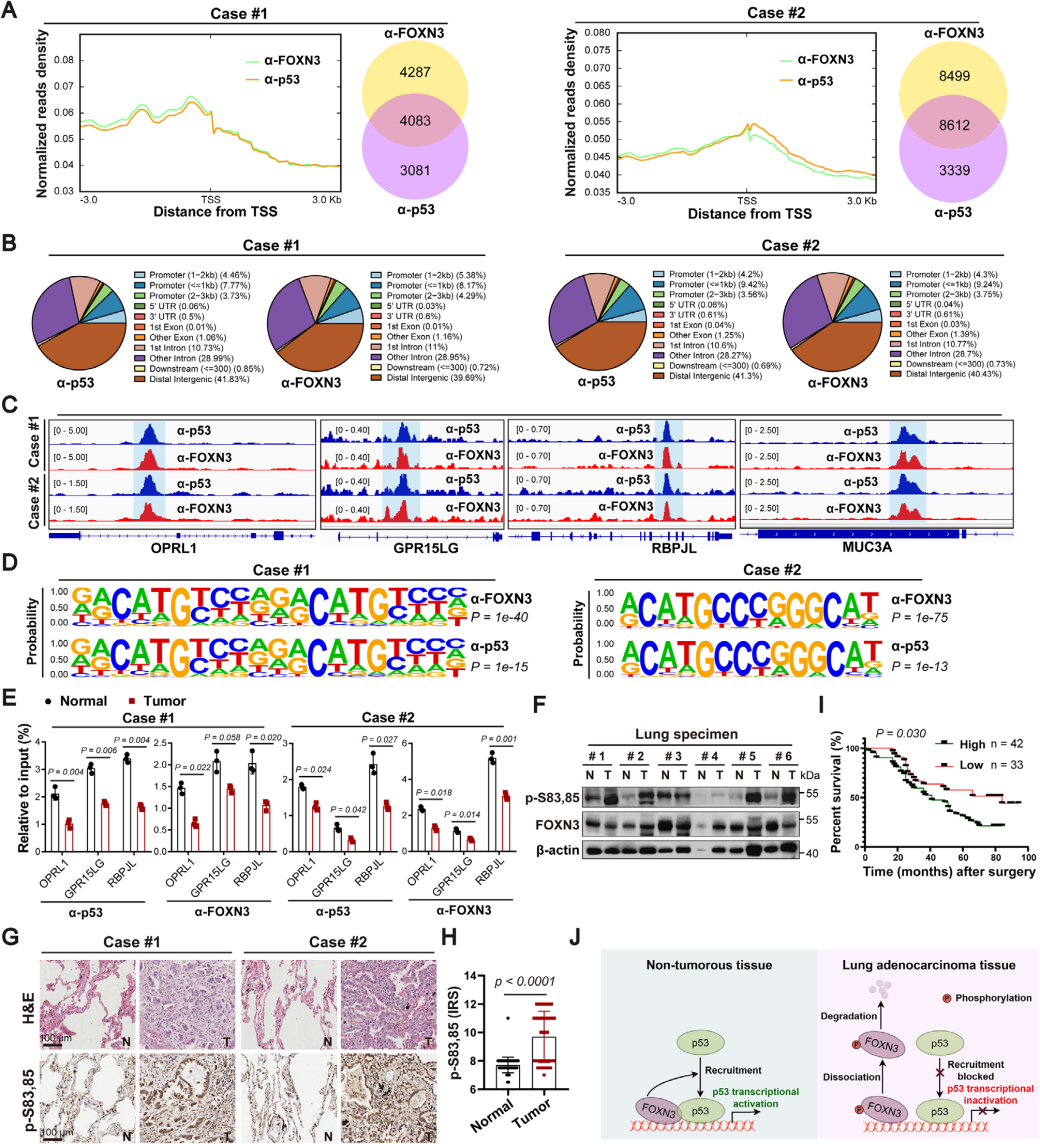
FOXN3‐mediated p53 transcriptional activity is closely associated with the prognosis of patients with clinical LUAD. (A) Density distributions (left panel) of FOXN3 and p53 peaks at the transcription start site and Venn diagrams (right panel) illustrating the overlapping genes co‐targeted by FOXN3 and p53 in clinical lung tumor samples, based on CUT&Tag data analysis. (B) Genomic distribution of the transcriptional targets of FOXN3 and p53 in clinical tumor samples, as determined via CUT&Tag data analysis. (C) The binding profiles of FOXN3 and p53 to transcriptional targets of p53 in clinical lung tumor tissues are displayed. (D) The two representative p53 binding motifs that are specifically bound by FOXN3 are presented. (E) ChIP assays were conducted on four paired lung tumor tissues and adjacent normal tissues isolated from patients with LUAD to assess the binding affinity of FOXN3 and p53 to the transcriptional targets of p53. Additional paired lung tumor samples can be found in Figure S7B. (F) Western blot analysis was conducted on 16 paired clinical lung tumor tissues and adjacent normal tissues obtained from patients with LUAD. Additional paired lung tumor samples can be found in Figure S7C. (G,H) Anti‐phosphorylated S83 and S85 FOXN3 immunohistochemical staining (G) of representative two pairs of clinical lung tumor tissues and adjacent normal tissues obtained from patients with LUAD. The levels of FOXN3 S83 and S85 phosphorylation were quantified by the immunoreactive score (IRS) (H). (I) A Kaplan‒Meier plot of overall survival for patients with LUAD was generated, stratified by high IRS (> 8) and low IRS (≤ 8) FOXN3 S83 and S85 phosphorylation levels. (J) A schematic representation illustrating the regulation of p53 transcriptional activity by phosphorylated FOXN3. The data D was assessed via two‐tailed Student's *t*‐tests. All the data are shown as the means ± SD.

The functional implications of the FOXN3/p53 complex in regulating LUAD in mice prompted us to investigate its clinical relevance. To address this, we first conducted CUT&Tag analysis on tumor tissues isolated from two patients with LUAD to determine whether FOXN3 has a genomic occupancy similar to that of p53. Remarkably, consistent with the in vitro cellular data, analyses of clinical lung tumor tissues revealed a substantial number of genes co‐targeted by FOXN3 and p53, with 4083 identified in Patient 1 and 8612 identified in Patient 2 (Figure 7A). These co‐targeted genes presented similar genomic landscapes and peak locations (Figure 7A,B), indicating a coordinated regulatory role of FOXN3 in p53‐mediated transcriptional regulation. Furthermore, among these co‐target genes, we selected and analyzed eight genes with the most abundant peaks via the IGV tool to illustrate their peak locations. In line with our CUT&Tag data from A549 cells presented in Figure 1, FOXN3 and p53 demonstrated strong colocalization in clinical tumor tissues (Figure 7C and Figure S7A). Analysis of the binding motifs revealed that both FOXN3 and p53 specifically bound to the p53‐specific binding elements (Figure 7D), indicating a potentially strong biochemical and functional connection. Furthermore, we performed ChIP assays coupled with quantitative PCR analyses on four paired clinical lung adenocarcinoma samples to evaluate the binding ability of the FOXN3/p53 complex at the aforementioned genes. Notably, our data clearly indicated that both FOXN3 and p53 occupancy at these gene loci were significantly reduced in tumor tissues compared to the paired adjacent normal samples (Figure 7E and Figure S7B). This suggests the crucial role of FOXN3 in the transcriptional inactivation of p53 during lung tumorigenesis.

On the basis of the critical role of FOXN3 S83 and S85 phosphorylation in its association with chromatin and activation of p53 signaling, we examined the expression patterns of S83 and S85 phosphorylation in tumor tissues and corresponding adjacent nontumorous tissues. Notably, WB analysis revealed that the levels of FOXN3 S83 and S85 phosphorylation in tumor samples were dramatically increased compared with those in paired normal tissues, despite a moderate decrease in total FOXN3 levels (Figure 7F and Figure S7C). To validate this finding, we performed immunohistochemical staining on 75 paired clinical tumor samples with an antibody against phosphorylated FOXN3, which further confirmed a marked increase in FOXN3 S83 and S85 phosphorylation in tumor tissues relative to adjacent nontumorous tissues (Figure 7G,H). Additionally, we investigated the survival relevance of FOXN3 phosphorylation at S83 and S85. Our data revealed that the high‐expression group presented shorter survival times (Figure 7I), indicating a poor prognosis associated with FOXN3 S83 and S85 phosphorylation. These collective observations provide strong evidence that FOXN3 is clinically relevant to the development of LUAD, potentially through its phosphorylation at S83 and S85.

## 3 Discussion

As a pivotal tumor suppressor gene, p53 plays an essential role in regulating various cancers, including LUAD. In this study, we identified the transcription factor FOXN3 as a critical cofactor of p53 that significantly contributes to suppressing LUAD progression by enhancing p53 transcriptional activity. Our CUT&Tag data revealed that FOXN3 and p53 exhibit striking genome‐wide co‐localization at gene promoters and share peak distributions in both lung cancer cell lines and clinical tumor tissues from patients with LUAD, suggesting a functional interaction in the regulation of transcriptional responses. Importantly, we characterized FOXN3 as a trans‐activator that directly interacts with p53, facilitates its recruitment to target gene regulatory elements, and thereby promotes p53 transcriptional activation (Figure 7J). Loss of FOXN3 leads to a significant reduction in p53 chromatin occupancy, resulting in repressed p53 transcriptional activity. Although FOXN3 has been previously characterized as a transcriptional repressor that interacts with various cofactors to mediate repressive functions [27, 28, 29], our study reveals that FOXN3 can also function as a transcriptional activator by enhancing p53 activity through its transactivation effects. These findings suggest that FOXN3 may exert varying regulatory influences depending on the specific biochemical context. Intriguingly, our investigations show that nearly all previously reported DNA‐binding‐deficient point mutants of p53, located within its DNA‐binding domain, exhibit a significantly reduced affinity for FOXN3. Furthermore, our deletion‐mapping assay demonstrated that FOXN3 specifically binds to the DNA‐binding domain of p53. Collectively, these findings suggest that FOXN3 is a crucial mediator in modulating p53's interaction with DNA through direct binding, which may be a prerequisite for p53's DNA binding capability. Additionally, the forkhead DNA‐binding domain of FOXN3 is essential for its association with p53. Overall, these results support a model in which DNA‐bound FOXN3 facilitates the formation of a p53/FOXN3/DNA complex, thereby promoting the transcriptional activation of p53. Investigating the structural basis of this complex could provide valuable insights for future studies aimed at restoring p53's DNA‐binding capacity and enhancing its transcriptional activity.

Our in vivo functional studies employing conditional KO of FOXN3 demonstrated a marked increase in the progression of primary lung tumors driven by the Kras G12D mutation following the loss of FOXN3. These findings suggest that the FOXN3‐p53 axis may coordinate with Kras signaling to regulate the pathological processes of LUAD, highlighting its potential as a promising therapeutic target for this aggressive disease.

In the present study, we provide strong evidence that FOXN3 acts as a tumor suppressor gene in the progression of LUAD through its positive regulation of p53 signaling. However, importantly, FOXN3 may have opposing functions in the context of other cancers because of different regulatory mechanisms. For example, one study reported that FOXN3 physically interacts with the NEAT1/SIN3A complex to promote epithelial‐mesenchymal transition (EMT) and metastasis in breast cancer [47]. Conversely, another study revealed that FOXN3 suppresses the growth and invasion of papillary thyroid cancer by inactivating the Wnt/β‐catenin pathway [48]. These contrasting roles of FOXN3 in various cancer types highlight the complexity of its function, which appears to be highly context‐dependent. In our study, we demonstrated that FOXN3 serves as a tumor suppressor regulator, specifically in LUAD progression. This multifaceted role of FOXN3 across different cancers warrants further investigation to better understand its underlying mechanisms and potential therapeutic implications. Future studies are essential to elucidate how FOXN3 influences cancer biology in diverse contexts and to explore its potential as a target for cancer treatment.

Our previous reports established that FOXN3 can be phosphorylated by p38 kinase at its S83 and S85 residues. This phosphorylation event leads to the dissociation of FOXN3 from chromatin and subsequent rapid degradation [38]. On the basis of these findings, we hypothesized that phosphorylation at S83 and S85 might play a role in regulating p53 transcriptional activity and the progression of LUAD. Our results demonstrate that ablation of FOXN3 phosphorylation at S83 and S85 enhances its association with chromatin and increases p53 transcriptional activity (Figure 7J). Moreover, the loss of the forkhead DNA‐binding domain of FOXN3 significantly diminished its binding to p53, consistent with previous reports indicating that the forkhead domain not only serves as a DNA‐binding domain but also functions as a transactivation domain [49]. These findings indicate that the positive regulation of p53 signaling by FOXN3 is a chromatin‐dependent biochemical event that relies on its binding to chromatin. Functional studies further revealed that genetic ablation of FOXN3 phosphorylation at these sites significantly impairs lung cancer cell survival, invasion, and tumor formation by activating p53 signaling. Our previous research has established that p38 is the direct kinase responsible for phosphorylating S83 and S85, which subsequently leads to FOXN3 degradation. Additionally, numerous studies have indicated that p38 plays a crucial role in the phosphorylation of p53, thereby regulating its transcriptional activity [50, 51, 52]. While our current in vitro findings underscore the importance of FOXN3 phosphorylation at S83 and S85 in modulating p53 transcriptional activity, questions remain regarding whether p38 contributes to FOXN3‐mediated p53 activation or if other kinases may directly phosphorylate S83 and S85, influencing this biochemical process in vivo. These inquiries are of significant interest. To address these questions comprehensively, future research may involve developing a knockout mouse model for p38.

Importantly, we assessed the levels of S83 and S85 phosphorylation in clinical samples from patients with LUAD to investigate the clinical relevance of these phosphorylation events in the pathological process of LUAD. Our data revealed a significant increase in S83 and S85 phosphorylation in tumor tissues compared to paired adjacent normal tissues. Additionally, survival analysis of these patients revealed that higher levels of FOXN3 phosphorylation at S83 and S85 are associated with poorer prognosis, suggesting that these phosphorylation events could serve as novel therapeutic targets for managing LUAD. This research not only enhances our understanding of the molecular mechanisms underlying tumor progression but also paves the way for future investigations into targeted cancer therapies aimed at modulating FOXN3 phosphorylation and its interactions with p53 signaling.

## 4 Materials and Methods

### 4.1 Cell Culture and Isolation

Dulbecco's Modified Eagle Medium (DMEM) was used to culture HEK293T, NIH3T3, and mouse embryonic fibroblasts (MEFs), whereas F‐12K medium was used for A549 cells. Each growth medium was enriched with 10% fetal calf serum (Gibco) and 1% penicillin/streptomycin. All cell lines were maintained at 37°C under a humidified, 5% CO_2_ atmosphere. HEK293T, NIH3T3, and A549 cells were obtained from the American Type Culture Collection (ATCC), while MEFs were isolated from WT or *Foxn3^KI/KI^* mouse embryos harvested at embryonic day 13.5 (E13.5).

### 4.2 Antibodies and Chemical Reagents

The following primary antibodies were used: anti‐p53 (10442‐1‐AP, Proteintech), anti‐HA (51064‐2‐AP, Proteintech), anti‐p21 (10355‐1‐AP, Proteintech), anti‐Bax (50599‐2‐Ig, Proteintech), anti‐MDM2 (86934, Cell Signaling Technology), anti‐His (66005‐1‐Ig, Proteintech), anti‐GST (10000‐0‐AP, Proteintech), anti‐FOXN3 (25399‐1‐AP, Proteintech), anti‐FOXN3 (A15039, ABclonal), anti‐Myc (16286‐1‐AP, Proteintech), anti‐Flag (20543‐1‐AP, Proteintech), anti‐GAPDH (2118, Cell Signaling Technology), anti‐rabbit IgG (30000‐0‐AP, Proteintech), anti‐FOXN3 (ab129453, Abcam) and anti‐p21 (YT3497, Immunoway). A polyclonal phosphospecific antibody targeting FOXN3 S83/S85 was developed by Dia‐An Biotechnology Co., Ltd. (Wuhan, China) using the phosphopeptides (VLRSpVSpPVQ) as the immunogen. CoraLite594–conjugated Goat Anti‐Rabbit (SA00013‐4, Proteintech) and CoraLite488‐conjugated Goat Anti‐Mouse (SA00013‐1, Proteintech) were used for immunofluorescence assay. Anti‐DYKDDDDK magnetic beads (A36798) and anti‐HA magnetic beads (88836) were purchased from Thermo Fisher Scientific. The chemical reagent Doxorubicin (HY‐15142) was from MedChemExpress, and Cisplatin (ST1164) was sourced from Beyotime.

### 4.3 Plasmid Constructions

Flag‐tagged FOXN3 and its S83,85A mutant were generated as previously described [46]. HA‐tagged p53 was cloned into a pCMV6 vector using reverse transcription PCR. The Flag‐tagged FOXN3 S83,85A mutant was introduced via site‐directed mutagenesis. Additionally, Flag‐tagged truncated mutants of FOXN3 (ΔForkhead, ΔNTD, ΔCTD) were generated as previously described [46]. All constructs were verified by DNA sequencing.

### 4.4 Lentivirus and Adeno‐Associated Virus

A pLV‐EF1α‐based core vector expressing the protein of interest was co‐transfected into HEK293T cells, together with the psPAX2 packaging and pMD2.G envelope plasmids, to generate lentiviral particles. Lentiviral infection was performed according to previously established protocols [53]. The adeno‐associated virus encoding Cre recombinase was obtained from OBIO Company (Shanghai, China).

### 4.5 RNAi Knockdown

For gene silencing, a pHBLV‐U6‐based lentiviral vector was used to express shRNAs. The shRNA sequences used were: shFOXN3, 5’‐GCCTGACATCCGATTAGAA‐3’. The siRNA sequences used in this study were as follows: siFOXN3 #1, 5’‐ GCCTGACATCCGATTAGAA ‐3’; siFOXN3 #2, 5’‐ AGACGATGACCTCGACTTT ‐3’. For NIH3T3 cells, the target sequences of the siRNA are: siFOXN3 #1, 5’‐CGACGACCACTATGAGTTT‐3’; siFOXN3 #2, 5’‐GTACCTTCTTCAAGAGAAA‐3’.

### 4.6 Migration and Invasion Assays

Medium supplemented with 10% FBS of 500 µL was added to the lower chambers. For the migration assay, 1–4 × 10^4^ cells were resuspended in 200 µL of FBS‐free medium and seeded into the upper chambers without Matrigel. In the invasion assay, the same number of cells (1–4 × 10^4^) was added to the upper chambers that were coated with Matrigel. After approximately 48 h of incubation, 500 µL of 4% paraformaldehyde was added to the lower chambers to fix the cells on the upper membrane filter for 2 h. Following fixation, 500 µL of crystal violet was used to stain the cells. The migratory cells were then visualized using microscopy.

### 4.7 Apoptosis Assay

An Annexin V FITC Apoptosis Detection Kit (Beyotime, China) was used to conduct an apoptosis assay. Briefly, cells were grown to approximately 80%–90% confluency, then washed with PBS, harvested, centrifuged, and resuspended in 1× Annexin V binding solution to achieve a concentration of 1 × 10^6^ cells/mL. The cells were subsequently stained with Annexin V‐FITC and propidium iodide (PI) solution at room temperature in the dark for 15 min. After staining, the samples were analyzed by flow cytometry.

### 4.8 Cell Cycle Analysis

Cells were collected and fixed overnight at 4°C using 70% ethanol. Following the manufacturer's instructions (Beyotime, China), the fixed cells were washed and then incubated with RNase A and propidium iodide (PI) for 30 to 60 min at room temperature (25°C) in the dark. The samples were subsequently analyzed by flow cytometry. Cell cycle distribution was assessed with FlowJo 7.6 software.

### 4.9 Immunoprecipitation

Cells were disrupted using a lysis buffer composed of 50 mm Tris‐Cl (pH 7.4), 250 mm NaCl, 1% Triton X‐100, and 1 mm EDTA, which was further supplemented with protease and phosphatase inhibitor cocktails. Following lysis, the samples were incubated with the designated antibodies to perform immunoprecipitation. The complete protocol followed a previously described method [54].

### 4.10 Stepwise Fractionation Assay

Briefly, 1 × 10^7^ cells were first subjected to swelling in buffer A (10 mm HEPES pH 7.9, 1.5 mm MgCl_2_, 10 mm KCl, 1 mm DTT, and 0.5 mm PMSF). The swollen cells were then extracted with buffer A supplemented with 1% NP‐40. Following this, the isolated nuclei underwent two further rounds of extraction using a low‐salt formulation of buffer A containing 0.5% NP‐40 and 75 mm NaCl. The supernatants collected from all three extraction steps were combined and termed the low‐salt fraction (LSF). The nuclei remaining after the low‐salt extractions were then lysed in SDS‐loading buffer at a volume equal to that of the LSF; this lysate was referred to as the low‐salt extracted nuclei (LSEN) and used for further immunoblot analysis.

### 4.11 Mouse Lung Tumorigenesis Models

In the loxP‐stop‐loxP (LSL) Kras G12D mutation‐driven primary lung tumorigenesis model, six‐week‐old *Kras^G12D/+^* mice were intratracheally administered AAV‐Cre (5 × 10^8^ PFU) for 18 weeks to induce lung tumorigenesis. In the A549 cell transplantation lung tumor model, 1 × 10^6^ A549 cells stably expressing the indicated proteins or shRNAs via lentiviral infection were transplanted into mice by tail vein injection. After six weeks of transplantation, the mice were sacrificed for subsequent tumorigenesis studies.

### 4.12 Animal Studies

This study involved C57BL/6 mice aged 8–10 weeks. The *Foxn3^LSL^* KI mice, which carry a floxed stop cassette at the Hipp11 locus [40], were generously provided by Dr. Yinming Liang at Xinxiang Medical University. For the FOXN3 S83,85A knock‐in mice, the S83 and S85 residues of FOXN3 were both mutated to alanine on a C57BL/6 background [46]. The point mutations were introduced through CRISPR/Cas9‐mediated knock‐in experiments, and the detailed protocol was performed as previously described [38]. The *Foxn3^flox/flox^* mice were generated using the CRISPR/Cas9 gene‐editing system, as previously outlined [38]. The *Kras^G12D/+^* strain (C57BL/6‐*Kras^em4(LSL‐G12D) Smoc^*, RRID: IMSR_NM‐KI‐190003) were obtained from Shanghai Model Organisms. Genotyping was confirmed through PCR analysis. All the animal studies were approved by the Institutional Review Board of Bengbu Medical University (Approval No. 2021–082). The study was compliant with all relevant ethical regulations regarding animal research.

### 4.13 Histological Assays

Mouse lungs were fixed in 4% paraformaldehyde, then embedded in paraffin and sectioned at a thickness of 4 µm. The sections were subsequently subjected to hematoxylin and eosin (H&E) staining or immunohistochemical staining analysis. Detailed procedures were performed as previously described [38].

### 4.14 High‐Throughput mRNA Sequencing

mRNA sequencing analysis was performed by GENEWIZ (Suzhou, China). Briefly, total RNA isolated using TRIzol reagent was subjected to library construction following the standard Illumina protocol. A paired‐end RNA‐seq approach was employed to sequence the libraries on the Illumina NovaSeq 6000 platform, and data analysis was conducted as previously described [55]. Raw data are available in the Sequence Read Archive (SRA) with accession numbers SRR30891916, SRR30891917, SRR30891918, SRR30891919, SRR30891920 and SRR30891921.

Link to the raw data: (https://www.ncbi.nlm.nih.gov/biosample?LinkName = bioproject_biosample_all&from_uid = 1166715).

### 4.15 Quantitative Real‐Time PCR Analysis

Total RNA (1 µg) was extracted from cells using TRIzol reagent and reverse transcribed following the manufacturer's instructions for the reverse transcription kit from Applied Biological Materials Inc. (abm). Quantitative PCR (qPCR) was performed using a qPCR Master Mix (also from abm), as previously described [54]. Relative gene expression levels were calculated using the 2^‐ΔΔCT^ method. The primer sequences used for gene amplification are listed in Tables S1 and S2.

### 4.16 Luciferase Assay

A p53 response element‐driven firefly luciferase reporter and a Renilla luciferase reporter plasmid were cotransfected with the indicated plasmids into cells, and luminescence was analyzed to assess p53 transcriptional activity.

### 4.17 CUT&Tag Assay

CUT&Tag assay was performed using a NovoNGS CUT&Tag High‐Sensitivity Kit. Cells were lysed by incubation on ice for 10 min, followed by centrifugation to isolate intact nuclei. After cross‐linking with formaldehyde for 2 min, the nuclei were quenched with glycine. Concanavalin A‐coated magnetic beads were then added and incubated for 15 min. The unbound supernatant was removed, and the beads were incubated with the primary antibody overnight at 4°C, followed by incubation with a secondary antibody for 1 h. After washing, the nuclei were incubated with the pA‐Tn5 adapter complex for 1 h, and then with a Mg^2^^+^‐containing tagmentation buffer. Subsequently, samples were treated with SDS overnight at 37°C. DNA purification, library preparation, and sequencing analysis were carried out according to previously reported procedures [40]. The raw CUT&Tag data for lung tumor tissues had been deposited in the Sequence Read Archive (SRA) database under accession numbers SRR30892124, SRR30892125, SRR30892126, SRR30892127. The raw CUT&Tag data for A549 cells are available in the SRA database under accession numbers SRR30892128, SRR30892129 and SRR30892130.

Link to the raw data:

(https://www.ncbi.nlm.nih.gov/biosample?LinkName = bioproject_biosample_all&from_uid = 1166715).

### 4.18 Chromatin Immunoprecipitation (ChIP) Assay

The 1×10^6^ cells were crosslinked with 1% formaldehyde for 10 min, after which the reaction was quenched with glycine. The cells were then lysed with a lysis buffer, followed by sonication to obtain fragmented DNA ranging from 100 to 500 bp. Following sonication, the cell lysates were centrifuged, and the resulting supernatant was subjected to immunoprecipitation using the indicated primary antibodies. Quantitative PCR analysis was subsequently performed. Detailed procedures were carried out as previously reported [56]. The primer sequences used for quantitative PCR analysis are listed in Table S3.

### 4.19 Patient Samples

Lung samples from patients with LUAD were obtained in accordance with research ethics board approval from Bengbu Medical University (Approval No. 2021‐014). The samples collected included both tumor tissue and corresponding adjacent normal areas. The samples were subsequently subjected to H&E staining or immunohistochemical staining using antibodies against phosphorylated S83 and S85 of FOXN3, following previously described protocols [38]. The detailed clinical information of the patients is shown in Table S4.

### 4.20 Statistical Analysis

All the statistical analyses were conducted via GraphPad Prism 8 software. Two‐tailed Student's *t*‐test was used to compare two groups, whereas one‐way ANOVA followed by Tukey's post hoc test was used for comparisons involving three or more groups. A *p*‐value of less than 0.05 was considered statistically significant. All the data were obtained from a minimum of three independent experiments, unless otherwise indicated, and are presented as the means ± SD.

## Author Contributions

X.Z., W.L., S.G.Z., C.Z., and H.Z. conceived and designed the project. Jinjin Y., Jingyu Y., S.W., and X.H. performed most of the experiments. X.H., Jinjin Y., and N.W. performed data analysis. Y.Z., J.Z., and Chengling Z. were responsible for the collection of clinical samples and subsequent data analysis. X.Z. drafted the manuscript. All authors reviewed, provided feedback, and agree on the authorship claims.

## Ethics Statement

The animal experiments were approved by the Institutional Review Board of Bengbu Medical University (Approval No. 2021–082) and conducted in compliance with all relevant ethical regulations regarding animal research. All lung samples from patients with LUAD were obtained with approval from the Research Ethics Board of Bengbu Medical University (Approval No. 2021‐014). Written informed consent was obtained from all participants prior to the start of the study.

## Conflicts of Interest

The authors declare no conflicts of interest.

## Supporting information




**Supporting File**: advs76449‐sup‐0001‐SuppMat.docx.

## Data Availability

The raw RNA sequencing data have been deposited in the Sequence Read Archive (SRA) database with accession numbers SRR30891916, SRR30891917, SRR30891918, SRR30891919, SRR30891920 and SRR30891921. The raw data for CUT&Tag have been deposited in the Sequence Read Archive (SRA) database with accession numbers SRR30892124, SRR30892125, SRR30892126, SRR30892127. The raw data for the CUT&Tag analysis on A549 cells have been deposited in the SRA database with accession numbers SRR30892128, SRR30892129 and SRR30892130.
