# Supplementary material for: Modulation of Lung Adenocarcinoma by Phosphorylated FOXN3‐Mediated Transcriptional Inactivation of p53
Source: Adv Sci (Weinh). 2026 Jul 7;13(54):e76449. doi: 10.1002/advs.76449 (PMC13339424; doi:10.1002/advs.76449)
Supplement: Supplementary file 1 [file ADVS-13-e76449-s001.docx]

**Supplementary information**

**Modulation of Lung Adenocarcinoma by Phosphorylated FOXN3-Mediated Transcriptional Inactivation of p53**

**Figure S1–S7**

**Fig. S1.** FOXN3 interacts with and colocalizes with p53 in lung cancer cells.

**Fig. S2.** Loss of FOXN3 does not affect the protein level of p53 but suppresses the expression of p53 downstream target genes.

**Fig. S3.** FOXN3 regulates the transcriptional recruitment of p53 without affecting its nuclear translocation.

**Fig. S4.** The loss of FOXN3 promotes lung cancer cell survival, invasion, and tumor formation.

**Fig. S5.** The S83,85D mutant mimicking phosphorylation of FOXN3 impeded the transcriptional activation of p53 via impaired interaction.

**Fig. S6.** Disruption of FOXN3 phosphorylation at S85 and S85 suppresses lung tumor formation.

**Fig. S7.** FOXN3 phosphorylation-mediated p53 transcriptional activity is associated with clinical lung adenocarcinoma.

**Table S1–S4**

**Table S1.** The primer sequences for quantitative PCR analysis targeting human genes

**Table S2.** The primer sequences for quantitative PCR analysis targeting mouse genes

**Table S3.** The primer sequences for quantitative PCR analysis of mouse genes

**Table S4.** Clinical information on patients with LUAD

Fig. S1

**Fig. S1. FOXN3 interacts with and colocalizes with p53 in lung cancer cells. Related to Fig. 1**

**(A)** An immunofluorescence assay was performed in A549 cells to assess the colocalization of Flag-tagged FOXN3 and HA-tagged p53.

**(B)** A deletion-mapping assay was performed in HEK293T cells to define the region within FOXN3 associated with p53.

**(C and D)** KEGG analysis highlighting the co-enriched signaling pathways identified from the anti-FOXN3 (C) and anti-p53 (D) CUT&Tag analyses in A549 cells.

Fig. S2

**Fig. S2. Loss of FOXN3 does not affect the protein level of p53 but suppresses the expression of p53 downstream target genes. Related to Fig. 2**

**(A)** WB analysis was performed in A549 cells with or without FOXN3 knockdown to examine the protein levels of representative downstream target genes of p53. The cells were treated with Dox (10 μM, 3 h) prior to collection.

**(B)** WB analysis was performed in NIH3T3 cells with or without FOXN3 knockdown to examine the protein levels of representative downstream target genes of p53. The cells were treated with Dox (2 μM, 24 h) prior to collection.

**(C)** A qPCR assay was performed in NIH3T3 cells with or without FOXN3 knockdown to examine the RNA levels of representative downstream target genes of p53. The cells were treated with Dox (2 μM, 24 h) prior to collection.

**(D)** A luciferase assay was conducted in HEK293T cells to assess the effect of FOXN3 overexpression on p53 transcriptional activity. A luciferase reporter driven by a promoter containing a p53 response element was co-transfected with Flag-tagged FOXN3 into the cells.

**(E)** WB analysis was performed in WT and FOXN3-KO A549 cells to examine the protein levels of p53. The cells were treated with Dox (10 μM, 3 h) prior to collection.

**(F)** WB analysis was conducted in A549 cells to detect the effect of FOXN3 knockdown on p53 protein levels. The cells were treated with Dox (10 μM, 3 h) prior to collection.

Data B was assessed via two-tailed Student's *t*-tests and data D was assessed via one-way ANOVA. All the data are shown as the means ± SD.

Fig. S3

**Fig. S3. FOXN3 regulates the transcriptional recruitment of p53 without affecting its nuclear translocation. Related to Fig. 2**

**(A)** Cytoplasmic and nuclear fractionation was conducted in A549 cells, with or without Dox (10 μM, 3 h) treatment, to evaluate the impact of FOXN3 knockdown on the subcellular distribution of p53.

**(B)** Cytoplasmic and nuclear fractionation analysis was conducted in NIH3T3 cells, with or without Dox (10 μM, 6 h) treatment, to evaluate the impact of FOXN3 knockdown on the subcellular distribution of p53.

**(C)** An immunofluorescence assay was performed in A549 cells to assess the impact of FOXN3 knockdown on the nuclear translocation of exogenous HA-tagged p53. The cells were treated with Dox (10 μM, 3 h) before collection.

Fig. S4

**Fig. S4. The loss of FOXN3 promotes lung cancer cell survival, invasion, and tumor formation. Related to Fig. 3**

**(A-C)** H&E staining analysis (A) was performed on lung sections from NOD/SCID mice that were administered either FOXN3-depleted A549 cells or mock control-treated A549 cells via tail-vein injection. The lung tumor burden was quantified using ImageJ software (B). The knockdown efficiency of FOXN3 in A549 cells was confirmed by WB analysis (C). FOXN3 silencing was achieved via lentiviral infection of A549 cells.

**(D)** The viability of NIH3T3 cancer cells was assessed using a CCK-8 assay, both with and without FOXN3 knockdown.

**(E)** An apoptosis assay was conducted in NIH3T3 cells to investigate the effect of FOXN3 knockdown on NIH3T3 cell apoptosis. The cells were treated with Dox (2 μM, 24 h) prior to collection.

**(F)** Cell cycle analysis was conducted in NIH3T3 cells to investigate the effect of FOXN3 knockdown on NIH3T3 cell cycle progression. The cells were treated with Dox (500 nM, 6 h) prior to collection.

**(G and H)** Invasion (G) and migration (H) assays were conducted on NIH3T3 cells treated with Dox (500 nM, 6 h), with or without FOXN3 knockdown, to evaluate the effect of FOXN3 on the invasive capacity of these cells. The cells were quantified using ImageJ software. Scale bar, 100 μm

The data B and E-H were assessed via two-tailed Student’s *t*-tests, and the data D was assessed via two-way ANOVA. All the data are presented as the means ± SD.

Fig. S5

**Fig. S5. The S83,85D mutant mimicking phosphorylation of FOXN3 impeded the transcriptional activation of p53 via impaired interaction. Related to Fig. 5**

**(A)** A Co-IP assay was performed in *Foxn3^−/−^* A549 cells expressing Flag-tagged WT FOXN3, along with its S83,85D mutants, to assess their interactions with p53. The cells were treated with Dox (10 μM, 3 h) prior to collection.

**(B)** qPCR analysis was conducted on *Foxn3^−/−^* A549 cells infected with a lentivirus expressing flag-tagged WT or S83,85D mutant of FOXN3 to assess the impact of mimicking FOXN3 phosphorylation on the RNA levels of p53 target genes. The cells were treated with Dox (10 μM, 3 h) prior to collection.

The data B was assessed via a two-tailed Student's *t*-test. All the data are shown as the means ± SD.

Fig. S6

**Fig. S6. Disruption of FOXN3 phosphorylation at S85 and S85 suppresses lung tumor formation. Related to Fig. 6**

**(A-C)** H&E staining analysis (A) was performed on lung sections from NOD/SCID mice that were transplanted either A549 cells overexpressing WT FOXN3 or A549 cells overexpressing the S83,85A mutant via tail-vein injection. The lung tumor burden was quantified using ImageJ software (B). The expression levels of exogenously transfected WT FOXN3 or the S83,85A mutant in A549 cells were confirmed by WB analysis (C). Overexpression of WT FOXN3 or the S83,85A mutant in A549 cells was achieved via lentiviral infection.

The data B was assessed via two-tailed Student’s *t*-tests. All the data are presented as the means ± SD.

Fig. S7

**Fig. S7. FOXN3 phosphorylation-mediated p53 transcriptional activity is associated with clinical lung adenocarcinoma. Related to Fig. 7**

**(A)** The CUT&Tag analysis reveals the binding profiles of FOXN3 and p53 to the transcriptional targets of p53 in clinical lung tumor tissues.

**(B)** ChIP assays were conducted on two paired lung tumor tissues and adjacent normal tissues isolated from patients with lung adenocarcinoma to assess the binding affinity of FOXN3 and p53 to the transcriptional targets of p53.

**(****C)** WB analysis was performed on paired clinical lung tumor tissues and adjacent normal tissues obtained from patients with lung adenocarcinoma.

The data in B was assessed via two-tailed Student’s *t*-tests. All the data are presented as the means ± SD.

**Table S1**

The primer sequences for quantitative PCR analysis targeting human genes

| p21-F | TTCCCTCTTTGGCTCCCCTG |
| --- | --- |
| p21-R | CTCAGAGCCACCTGGAGCTG |
| MDM2-F | ACTCTCAGATGAAGATGATGA |
| MDM2-R | TTGCATGAAGTGCATTTCCAAT |
| Bax-F | CAAGCGCATCGGGGACGAAC |
| Bax-R | TGAAGTTGCCGTCAGAAAAC |
| FOXN3-F | CCTACGATGCCAGGCAGAAC |
| FOXN3-R | GTTCCAAGATCCAGTTGTAG |
| GAPDH-F | CATGAGAAGTATGACAACAGCCT |
| GAPDH-R | AGTCCTTCCACGATACCAAAGT |

**Table S2**

The primer sequences for quantitative PCR analysis targeting mouse genes

| p21-F | CCTGGTGATGTCCGACCTG |
| --- | --- |
| p21-R | CCATGAGCGCATCGCAATC |
| MDM2-F | CTCTGGACTCGGAAGATTACAGCC |
| MDM2-R | CCTGTCTGATAGACTGTCACCCG |
| Bax-F | GTTTCATCCAGGATCGAGCAG |
| Bax-R | CCCCAGTTGAAGTTGCCATC |
| FOXN3-F | TAGGAGTGTCAGTCCTGTGC |
| FOXN3-R | GAAGGCGCTTGGTCGGAGAG |
| GAPDH-F | AGGTCGGTGTGAACGGATTTG |
| GAPDH-R | TGTAGACCATGTAGTTGAGGTCA |

**Table S3**

The primer sequences for ChIP-quantitative PCR analysis

| p21-F | CTGGACTGGGCACTCTTGTC |
| --- | --- |
| p21-R | CTCCTACCATCCCCTTCCTC |
| MDM2-F | GGTTGACTCAGCTTTTCCTCTTG |
| MDM2-R | GGAAAATGCATGGTTTAAATAGCC |
| Bax-F | TAATCCCAGCGCTTTGGAAG |
| Bax-R | TGCAGAGACCTGGATCTAGCAA |
| OPRL1-F | CTTACCCATCCTCTCTCTTG |
| OPRL1-R | CCCCACAGCTTCCTCCCTAC |
| GPR15LG-F | TGCAGCCCCAGTGCCCACTC |
| GPR15LG-R | CCTCTCCTGCATCACAGCTG |
| RBPJL-F | CAGTTTGAAGGCTGATGCTG |
| RBPJL-R | GTCCTGTCCACTCCACCTTC |

**Table S4**

Clinical information on patients with LUAD

| **Patient** | **Gender** | **Age** | **Pathological type** |
| --- | --- | --- | --- |
| Case 1 | Female | 64 | LUAD |
| Case 2 | Male | 72 | LUAD |
| Case 3 | Male | 71 | LUAD |
| Case 4 | Female | 68 | LUAD |
| Case 5 | Female | 66 | LUAD |
| Case 6 | Female | 66 | LUAD |
| Case 7 | Female | 48 | LUAD |
| Case 8 | Male | 46 | LUAD |
| Case 9 | Male | 60 | LUAD |
| Case 10 | Female | 54 | LUAD |
| Case 11 | Female | 65 | LUAD |
| Case 12 | Female | 66 | LUAD |
| Case 13 | Female | 42 | LUAD |
| Case 14 | Male | 57 | LUAD |
| Case 15 | Male | 49 | LUAD |
| Case 16 | Male | 53 | LUAD |
| Case 17 | Male | 47 | LUAD |
| Case 18 | Male | 65 | LUAD |
| Case 19 | Male | 70 | LUAD |
| Case 20 | Male | 75 | LUAD |
| Case 21 | Male | 68 | LUAD |
| Case 22 | Male | 58 | LUAD |
| Case 23 | Male | 51 | LUAD |
| Case 24 | Female | 51 | LUAD |
| Case 25 | Female | 58 | LUAD |
| Case 26 | Female | 60 | LUAD |
| Case 27 | Female | 68 | LUAD |
| Case 28 | Male | 50 | LUAD |
| Case 29 | Female | 53 | LUAD |
| Case 30 | Male | 62 | LUAD |
| Case 31 | Male | 59 | LUAD |
| Case 32 | Male | 53 | LUAD |
| Case 33 | Female | 68 | LUAD |
| Case 34 | Female | 60 | LUAD |
| Case 35 | Male | 61 | LUAD |
| Case 36 | Male | 70 | LUAD |
| Case 37 | Female | 57 | LUAD |
| Case 38 | Female | 59 | LUAD |
| Case 39 | Male | 50 | LUAD |
| Case 40 | Female | 69 | LUAD |
| Case 41 | Female | 60 | LUAD |
| Case 42 | Female | 46 | LUAD |
| Case 43 | Female | 58 | LUAD |
| Case 44 | Male | 53 | LUAD |
| Case 45 | Female | 45 | LUAD |
| Case 46 | Female | 62 | LUAD |
| Case 47 | Male | 58 | LUAD |
| Case 48 | Female | 44 | LUAD |
| Case 49 | Male | 50 | LUAD |
| Case 50 | Female | 62 | LUAD |
| Case 51 | Male | 69 | LUAD |
| Case 52 | Male | 51 | LUAD |
| Case 53 | Male | 74 | LUAD |
| Case 54 | Female | 56 | LUAD |
| Case 55 | Male | 62 | LUAD |
| Case 56 | Male | 37 | LUAD |
| Case 57 | Male | 44 | LUAD |
| Case 58 | Male | 67 | LUAD |
| Case 59 | Female | 72 | LUAD |
| Case 60 | Female | 67 | LUAD |
| Case 61 | Male | 65 | LUAD |
| Case 62 | Female | 57 | LUAD |
| Case 63 | Male | 49 | LUAD |
| Case 64 | Male | 48 | LUAD |
| Case 65 | Female | 51 | LUAD |
| Case 66 | Female | 71 | LUAD |
| Case 67 | Male | 67 | LUAD |
| Case 68 | Male | 60 | LUAD |
| Case 69 | Female | 40 | LUAD |
| Case 70 | Female | 56 | LUAD |
| Case 71 | Male | 59 | LUAD |
| Case 72 | Male | 79 | LUAD |
| Case 73 | Female | 72 | LUAD |
| Case 74 | Female | 50 | LUAD |
| Case 75 | Male | 52 | LUAD |
